# The Value of Radiologic Interventions and ^18^F-DOPA PET in Diagnosing and Localizing Focal Congenital Hyperinsulinism: Systematic Review and Meta-Analysis

**DOI:** 10.1007/s11307-012-0572-0

**Published:** 2012-07-03

**Authors:** Björn A. Blomberg, Mateen C. Moghbel, Babak Saboury, Charles A. Stanley, Abass Alavi

**Affiliations:** 1 Perelman School of Medicine, University of Pennsylvania, 3400 Spruce Street, Philadelphia, PA USA; 2 Department of Radiology, Hospital of the University of Pennsylvania, Philadelphia, PA USA; 3 University Medical Center Utrecht, Utrecht University School of Medicine, Utrecht, The Netherlands; 4 Division of Endocrinology, Children’s Hospital of Philadelphia, Philadelphia, PA USA

**Keywords:** Congenital hyperinsulinism, Pancreatic venous sampling, Arterial stimulation venous sampling, ^18^F-DOPA PET, Positron emission tomography, Diagnosis

## Abstract

**Purpose:**

This systematic review and meta-analysis aimed to quantify the diagnostic performance of pancreatic venous sampling (PVS), selective pancreatic arterial calcium stimulation with hepatic venous sampling (ASVS), and ^18^F-DOPA positron emission tomography (PET) in diagnosing and localizing focal congenital hyperinsulinism (CHI).

**Procedures:**

This systematic review and meta-analysis was conducted according to the PRISMA statement. PubMed, EMBASE, SCOPUS and Web of Science electronic databases were systematically searched from their inception to November 1, 2011. Using predefined inclusion and exclusion criteria, two blinded reviewers selected articles. Critical appraisal ranked the retrieved articles according to relevance and validity by means of the QUADAS-2 criteria. Pooled data of homogeneous study results estimated the sensitivity, specificity, likelihood ratios and diagnostic odds ratio (DOR).

**Results:**

^18^F-DOPA PET was superior in distinguishing focal from diffuse CHI (summary DOR, 73.2) compared to PVS (summary DOR, 23.5) and ASVS (summary DOR, 4.3). Furthermore, it localized focal CHI in the pancreas more accurately than PVS and ASVS (pooled accuracy, 0.82 vs*.* 0.76, and 0.64, respectively). Important limitations comprised the inclusion of studies with small sample sizes, high probability of bias and heterogeneity among their results. Studies with small sample sizes and high probability of bias tended to overestimate the diagnostic accuracy.

**Conclusions:**

This systematic review and meta-analysis found evidence for the superiority of ^18^F-DOPA PET in diagnosing and localizing focal CHI in patients requiring surgery for this disease.

## Introduction

Congenital hyperinsulinism (CHI) represents a heterogeneous group of genetic disorders characterized by excessive insulin secretion for the level of glycaemia [1–3]. Up to 60 % of CHI patients fail to respond to dietary and medical measures and require surgical pancreatectomy to remain normoglycaemic [4–6]. The extent of pancreatectomy performed depends on the histopathological diagnosis: focal or diffuse disease [7]. Focal islet-cell hyperplasia can be cured by focal excision of the lesion [8, 9]. Diffuse β-cell hypersecretion requires near-total pancreatectomy, is only palliative, and may result in diabetes mellitus and/or exocrine pancreas insufficiency [4, 10, 11]. Therefore, it proves crucial to differentiate focal from diffuse disease before surgery is commenced [12]. Furthermore, preoperative localization of focal disease in the pancreas (i.e. distinction between pancreatic head, body, or tail) can help paediatric surgeons considerably in identifying the macroscopic indiscernible focal lesion from non-affected pancreatic tissue during curative surgery.

Because clinical presentation, genetic testing, and structural imaging methods such as ultrasound, computed tomography (CT), and magnetic resonance imaging (MRI) are usually non-discriminatory for the purpose of diagnosing and localizing focal CHI [1], interventional radiologic tests, such as pancreatic venous sampling (PVS) and combined selective pancreatic arterial calcium stimulation and hepatic venous sampling (ASVS), have been developed [13–16]. In addition, fluorine-18 labelled fluoro-l-DOPA (^18^F-DOPA) positron emission tomography (PET) imaging has been proposed as a technique to identify and localize focal disease [17, 18]. However, the diagnostic accuracy of each of these modalities has not been well established.

Therefore, the purpose of this article was to provide the best available evidence on the diagnostic accuracy of PVS, ASVS, and ^18^F-DOPA PET in diagnosing and localizing a focal subtype of CHI in patients requiring surgery for this disease by systematic review and meta-analysis of the literature.

## Materials and Methods

### Protocol

This systematic review and meta-analysis was conducted according to the PRISMA statement [19].

### Search Strategy

Using predefined search terms, the PubMed, EMBASE, SCOPUS and Web of Science electronic databases were systematically searched from inception to November 1, 2011. The search syntax is presented in Table 1. No search filters or language restrictions were imposed. A cross-reference check of included articles was used to identify additional articles missed by our search strategy.

**Table 1:** Search syntax

Database	Search syntax
PubMed	(“hyperinsulinism”[TIAB] OR “CHI”[TIAB] OR “nesidioblastosis”[TIAB] OR “islet cell”[TIAB] or “beta cell”[TIAB] OR “hypoglycemia”[TIAB] OR “PHHI”[TIAB] OR “HHI”[TIAB] OR “HI”[TIAB] OR “insulinoma”[TIAB] OR “hyperinsulinaemic”[TIAB] OR “hyperinsulinemic”[TIAB]) AND (“positron emission tomography”[TIAB] OR “PET”[TIAB] OR “^18^F-DOPA”[TIAB] OR “^18^F-fluoro-L-DOPA”[TIAB] OR “fluorine-18-L-3,4-dihydroxyphenylalanine”[TIAB] OR “venous sampling”[TIAB] OR “PVS”[TIAB] OR “calcium stimulation”[TIAB] OR “PACS”[TIAB] OR “ASVS”[TIAB] OR “vein catherization”[TIAB] OR “PPVC”[TIAB] OR “THPVS”[TIAB])
EMBASE	Replaced [TIAB] with :ab,ti
SCOPES	Replaced [TIAB] by search field: Article Title, Abstract, Keywords
Web of Science	Replaced [TIAB] by search field: Topic

### Eligibility Criteria

Two blinded reviewers assessed article eligibility using predefined inclusion and exclusion criteria. Relevant articles were included based on the following criteria: study domain—patients with CHI; index test—PVS, or ASVS, or ^18^F-DOPA PET; reference standard—histopathology obtained from surgery; study results—agreement between index and reference standard; study design—cross-sectional study format. In case of multiple studies reporting on an overlapping population, only the study with the largest patient population was included. Probability adjusted agreement (*κ* statistic) evaluated interrater agreement [20]. Discordant judgments were resolved by consensus discussion.

### Critical Appraisal

Two blinded reviewers appraised the relevance and validity of the selected papers by use of the revised Quality Assessment of Studies of Diagnostic Accuracy Included in Systematic Reviews (QUADAS-2) criteria [21]. The item ‘flow and timing’ was replaced by ‘missing data’ because the question “was there an appropriate interval between the index test and reference standard” was considered not meaningful since CHI is a long-standing genetic disease [22]. Furthermore, the item ‘blinding’ was presented separately to reflect its importance in bias assessment of diagnostic studies. Six authors were contacted to provide data on sufficient blinding protocols. Five authors responded and provided the requested data. The *κ* statistic evaluated interrater agreement. Discordant judgments were resolved by consensus discussion.

### Data Extraction

The following data were extracted from included articles: study design, study population, index test characteristics, reference standard characteristics, and localization accuracy. Furthermore, true positive, false-positive, true negative and false-negative rates were extracted and summarized in 2 × 2 contingency tables. Empty cells were filled with 0.5 events to allow calculation of the outcome measures of interest.

### Outcome Measures and Data Synthesis

Calculating the sensitivity, specificity, positive and negative likelihood ratio (LR), and the diagnostic odds ratio (DOR) along with 95 % confidence intervals determined the diagnostic accuracy of each index test in differentiating focal from diffuse CHI. The percentage of tests that localised the focal lesion in the correct area of the pancreas according to histopathology determined the localisation accuracy. Inability to detect focal CHI was considered a failed attempt to localise the focal lesion.

Heterogeneity was determined statistically by a Cochran *Q* (*χ* ^2^ statistic) test and the *Ι* ^2^ statistic for heterogeneity. An *Ι* ^2^ statistic of >25 % was considered evidence for clinically relevant heterogeneity [23]. In case of clinically relevant heterogeneity, outcome measures were estimated from studies of the highest scientific validity—that is, studies that are most likely to be free from bias. Data from statistically homogenous studies were pooled by means of a random-effects model (DerSimonian–Laird) to estimate outcome measures. Furthermore, pooled data was used to determine the area under the ROC curve (AUC) and the Q* statistic.

The presence of possible publication bias was evaluated graphically by drawing funnel plots for each outcome measure and statistically by means of Egger’s standard regression test. Statistical significance was claimed for *p* < 0.10 (two-tailed). We acknowledge that other factors, such as methodological heterogeneity or true study heterogeneity, could also introduce asymmetry in publication bias assessment [24].

Data analysis was performed using the publically available dedicated meta-analysis software-tool Meta-DiSc version 1.4 [25].

## Results

**Fig. 1:**
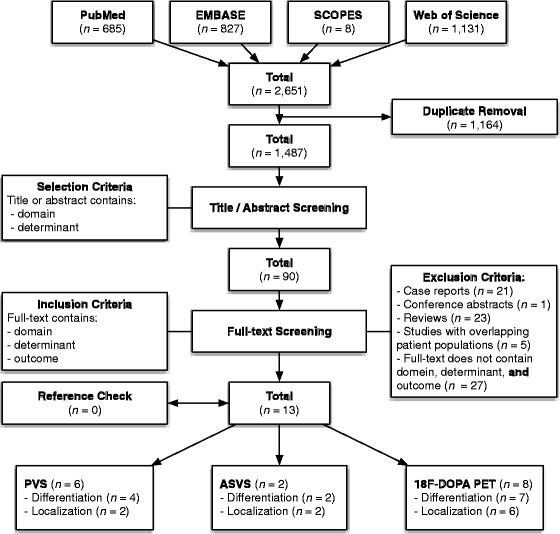
Flowchart of search strategy. Search performed on November 1st, 2011.

After adjusting for duplicates, our systematic search retrieved 1,487 possibly relevant articles. Thirteen articles (excluding five with overlapping patient populations [12, 14, 17, 18, 26]) met our predefined criteria and were selected for inclusion with very high interrater agreement (*κ* = 0.92) (Fig. 1).

Included trials were published between 1989 and 2011 and reported on a total of 415 patients with CHI (Table 2). All studies had a cross-sectional study design. The value of PVS was reported in six articles [7, 13, 27–30]. Of these, four studies reported data on differentiation accuracy, two reported data on localisation accuracy. The value of ASVS was reported in two studies [16, 31]. Both reported data on differentiation accuracy and localisation accuracy. Eight articles reported data on the value of ^18^F-DOPA PET [27, 29, 30, 32–36]. Of these, one reported data on localisation accuracy, two reported data on differentiation accuracy, and five articles reported both.

**Table 2:** Basic study characteristics of included articles in alphabetical order

Author (year)	Period	Patient characteristics		n^1^	Index test	n^2^	Reference standard	n^3^	Outcome	Study design
		Age at CHI diagnosis, months (range)	Female sex, %							
Barthlen et al. (2008) [32]	2005–2007	NR	NR	30	^18^F-DOPA PET/CT	30	Immunohistochemistry	11	Differentiation/localization	Cross-sectional
Brunnele et al. (1989) [13]	NR	NR	74	19	PVS	19	Histology	6	Differentiation	Cross-sectional
Capito et al. (2009) [27]	1995–2008	0 (0–9)	43	51	PVS	35	Histology	51	Localization	Cross-sectional
					^18^F-DOPA PET	16			Localization	
Chigot et al. (2001) [31]	NR	NR	NR	12	PVS	7	Histology	12	-	Cross-sectional
					ASVS	12			Differentiation/localization	
Crétolle et al. (2002) [28]	1983–2000	NR	69	45	PVS	48	Immunohistochemistry	45	Localization	Cross-sectional
de Lonlay et al. (1999) [7]	1985–1998	NR	53	52	PVS	45	Histology	52	Differentiation/localization	Cross-sectional
de Lonlay et al. (2006) [29]	NR	NR	NR	7	PVS	4	Immunohistochemistry	7	Differentiation	Cross-sectional
					^18^F-DOPA PET/MRI	7			Differentiation	
Hardy et al. (2007) [33]	2004–2007	NR	NR	50	^18^F-DOPA PET/CT	50	Immunohistochemistry	50	Differentiation/localization	Cross-sectional
Masue et al. (2011) [34]	2005–2010	(2–37)	47	17	ASVS	7	Histology	12	-	Cross-sectional
					^18^F-DOPA PET/CT	17			Differentiation	
Otonkoski et al. (2006) [35]	NR	(0–6)	NR	14	^18^F-DOPA PET/MRI	9	Histology	14	Differentiation/localization	Cross-sectional
Ribeiro et al. (2007) [30]	NR	(0–8)	NR	49	PVS	12	Immunohistochemistry	24	Differentiation	Cross-sectional
					^18^F-DOPA PET/MRI	49			Differentiation/localization	
Stanley et al. (2004) [16]	1998–2002	0 (0–14)	52	50	ASVS	50	Histology	50	Differentiation/localization	Cross-sectional
Zani et al. (2011) [36]	2006–2010	NR	42	19	^18^F-DOPA PET	19	Immunohistochemistry	19	Differentiation/localization	Cross-sectional

*CHI* congenital hyperinsulinism; *n* ^*1*^ number of patients; *n* ^*2*^ number of index tests performed; *n* ^*3*^ number of reference tests performed; *NR* not reported; *CT* computed tomography; *MRI* magnetic resonance imaging; ^*18*^ *F-DOPA PET* fluorine-18 l-3,4-dihydroxyphenylalanine positron emission tomography; *PVS* pancreatic venous sampling; *ASVS* selective pancreatic arterial calcium stimulation with hepatic venous sampling

Articles were ranked according to relevance and validity with good interrater agreement (κ = 0.82). Potential threats to relevance and scientific validity are summarized in Table 3.

**Table 3:** Assessment of methodological quality and applicability; in order of relevance and validity

Author (year)	Relevance			Validity					
	Domain	Index test	Reference standard	SDo	SIT	BRS	SRS	BIT	MD
de Lonlay et al. (1999) [7]	CHI	PVS	Histology	+	+	−	+	−	−
Brunelle et al. (1989) [13]	CHI	PVS	Histology	+	+	−	−	−	−
de Lonlay et al. (2006) [29]	CHI	PVS	Immunohistochemistry	−	−	−	+	−	−
Ribeiro et al. (2007) [30]	CHI	PVS	Immunohistochemistry	−	+	−	−	−	−
Capito et al. (2009) [27]	Focal CHI	PVS	Histology	+	+	−	+	−	+
Crétolle et al. (2002) [28]	Focal CHI	PVS	Immunohistochemistry	+	+	−	+	−	+
Stanley et al. (2004) [16]	CHI	ASVS	Histology	+	+	+	+	+	+
Chigot et al. (2001) [31]	CHI	ASVS	Histology	−	+	−	+	−	−
Hardy et al. (2007) [33]	CHI	^18^F-DOPA PET/CT	Immunohistochemistry	+	+	+	+	+	+
Zani et al. (2011) [36]	CHI	^18^F-DOPA PET/CT	Immunohistochemistry	+	+	?	+	?	+
de Lonlay et al. (2006) [29]	CHI	^18^F-DOPA PET/MRI	Immunohistochemistry	−	+	−	+	−	+
Barthlen et al. (2008) [32]	CHI	^18^F-DOPA PET/CT	Immunohistochemistry	−	+	+	+	+	−
Masue et al. (2011) [34]	CHI	^18^F-DOPA PET/CT	Histology	+	+	−	?	−	−
Ribeiro et al. (2007) [30]	CHI	^18^F-DOPA PET/MRI	Immunohistochemistry	−	+	−	?	−	−
Otonkoski et al. (2006) [35]	CHI	^18^F-DOPA PET	Histology	−	+	−	?	−	−
Capito et al. (2009) [27]	Focal CHI	^18^F-DOPA PET	Histology	+	+	−	+	−	+

*CHI* congenital hyperinsulinism; *PVS* pancreatic venous sampling; *ASVS* selective pancreatic arterial calcium stimulation with hepatic venous sampling; ^*18*^ *F-DOPA PET* fluorine-18 L-3,4-dihydroxyphenylalanine positron emission tomography; *CT* computed tomography; *MRI* magnetic resonance imaging; *SDO* standardized selection of domain; + standardized, transparent and reproducible, bias unlikely; − no standardization, bias likely; *SIT* standardized assessment of index test (IT); + standardized, transparent and reproducible, bias unlikely; − no standardization, bias likely; *BRS* blinding outcome of RS for assessor of IT; blinding, + bias unlikely; − no blinding, bias likely; *SRS* standardized assessment of reference standard (RS); + standardized transparent and reproducible, bias unlikely; − no standardization, bias likely; *BIT* blinding outcome of IT for assessor of RT; + blinding, bias unlikely; − no blinding, bias likely; *MD* missing data, including verification bias; + missing <10 %, bias unlikely; − missing data >10 %, bias likely

### Differentiation Accuracy

Table 4 shows the diagnostic accuracy of PVS, ASVS and ^18^F-DOPA PET differentiating focal and diffuse CHI.

**Table 4:** Diagnostic accuracy; in order of relevance and validity

Author (year)	Determinant	*n*	Sensitivity (95% CI)	Specificity (95% CI)	Positive LR (95% CI)	Negative LR (95% CI)	Diagnostic odds ratio (95% CI)
de Lonlay et al. (1999) [7]	PVS	45	0.90 (0.70, 0.99)	0.73 (0.52, 0.88)	3.3 (1.7, 6.4)	0.14 (0.04, 0.55)	32.1 (4.2, 126.6)
Brunelle et al. (1989) [13]	PVS	6	1.00 (0.29, 1.00)	1.00 (0.29, 1.00)	7.0 (0.51, 96.1)	0.14 (0.01, 1.96)	49.0 (0.74, 3237)
de Lonlay et al. (2006) [29]	PVS	4	1.00 (0.16, 1.00)	1.00 (0.16, 1.00)	5.0 (0.38, 66.0)	0.20 (0.02, 2.64)	25.0 (0.34, 1832)
Ribeiro et al. (2007) [30]	PVS	10	0.71 (0.29, 0,96)	1.00 (0.29, 1.00)	5.5 (0.40, 76.7)	0.36 (0.12, 1.07)	15.4 (0.56, 425.5)
Pooled estimate	PVS	65	0.87 (0.70, 0.96)	-	3.6 (1.99, 6.6)	0.23 (0.11, 0.50)	23.5 (6.09, 90.9)
Heterogeneity			*χ* ^2^: 2.68; *df* = 3	*χ* ^2^: 4.29; *df* = 3	*χ* ^2^: 0.51; *df* = 3	*χ* ^2^: 1.53; *df* = 3	*χ* ^2^: 0.18; *df* = 3
			*p* = 0.44; *Ι* ^2^: 0 %	*p* = 0.23; *Ι* ^2^: 30 %	*p* = 0.92; *Ι* ^2^: 0 %	*p* = 0.68; *Ι* ^2^: 0 %	*p* = 0.98; *Ι* ^2^: 0 %
Stanley et al. (2004) [16]	ASVS	48	0.69 (0.51, 0.83)	0.69 (0.39, 0.91)	2.2 (0.96, 5.2)	0.45 (0.25, 0.84)	4.9 (1.2, 14.7)
Chigot et al. (2001) [31]	ASVS	12	0.83 (0.36, 1.00)	0.33 (0.04, 0.78)	1.3 (0.64, 2.4)	0.50 (0.06, 4.2)	2.5 (0.16, 38.6)
Pooled estimate	ASVS	60	0.71 (0.55, 0.84)	-	-	0.46 (0.26, 0.82)	4.3 (1.3, 14.7)
Heterogeneity			*χ* ^2^: 0.59; *df* = 1	*χ* ^2^: 2.18; *df* = 1	*χ* ^2^: 1.46; *df* = 1	*χ* ^2^: 0.01; *df* = 1	*χ* ^2^: 0.19; *df* = 1
			*p* = 0.44; *Ι* ^2^: 0 %	*p* = 0.14; *Ι* ^2^: 54 %	*p* = 0.23; *Ι* ^2^: 31 %	*p* = 0.93; *Ι* ^2^: 0 %	*p* = 0.67; *Ι* ^2^: 0 %
Hardy et al. (2007) [33]	^18^F-DOPA PET/CT	50	0.75 (0.53, 0.90)	1.00 (0.87, 1.00)	40 (2.5, 628)	0.26 (0.14, 0.51)	150.9 (8.0, 2845)
Zani et al. (2011) [36]	^18^F-DOPA PET/CT	19	1.00 (0.77, 1.00)	1.00 (0.48, 1.00)	12 (0.8, 165)	0.04 (0, 0.56)	319.0 (5.6, 18145)
de Lonlay et al. (2006) [29]	^18^F-DOPA PET	7	1.00 (0.40, 1.00)	1.00 (0.29, 1.00)	7.2 (0.5, 97.8)	0.11 (0.01, 1.63)	63.0 (0.98, 4042)
Barthlen et al. (2008) [32]	^18^F-DOPA PET	11	0.90 (0.55, 1.00)	1.00 (0.03, 1.00)	3.5 (0.3, 39)	0.18 (0.03, 0.98)	19.0 (0.50, 719.8)
Masue et al. (2011) [34]	^18^F-DOPA PET/CT	12	0.67 (0.30, 0.93)	1.00 (0.29, 1.00)	5.2 (0.4, 72)	0.40 (0.16, 1.0)	13.0 (0.5, 330.5)
Ribeiro et al. (2007) [30]	^18^F-DOPA PET/MRI	24	0.93 (0.68, 1.00)	1.00 (0.66, 1.00)	18 (1.2, 271)	0.10 (0.02, 0.46)	183.7 (6.75, 4997)
Otonkoski et al. (2006) [35]	^18^F-DOPA PET	9	1.00 (0.48, 1.00)	1.00 (0.40, 1.00)	9.2 (0.7, 129)	0.09 (0.01, 1.3)	99.0 (1.62, 6053)
Pooled estimate	^18^F-DOPA PET	132	-	1.00 (0.93, 1.00)	9.49 (3.5, 26)	0.23 (0.14, 0.37)	73.2 (19.2, 279.7)
Heterogeneity			*χ* ^2^: 12.6; *df* = 6	*χ* ^2^: 0; *df* = 6	*χ* ^2^: 2.44; *df* = 6	*χ* ^2^: 6.09; *df* = 6	*χ* ^2^: 2.69; *df* = 6
			*p* = 0.06; *Ι* ^2^: 50 %	*p* = 1.00; *Ι* ^2^: 0 %	*p* = 0.88; *Ι* ^2^: 0 %	*p* = 0.41; *Ι* ^2^: 1 %	*p* = 0.85; *Ι* ^2^: 0 %

*n* number of study subject that underwent determinant and subsequent surgical intervention; *CT* computed tomography; *MRI* magnetic resonance imaging; *PVS* pancreatic venous sampling; *ASVS* selective pancreatic arterial calcium stimulation with hepatic venous sampling; ^*18*^ *F-DOPA PET* fluorine-18 l-3,4-dihydroxyphenylalanine positron emission tomography; *LR* likelihood ratio; *CI* confidence interval

**Fig. 2:**
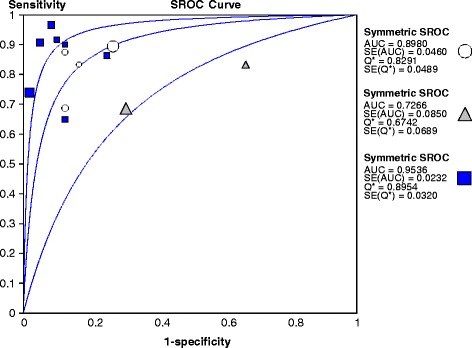
Summary ROC curve plotting the true positive rate (sensitivity) against the false-positive rate (1-specificity). Each *symbol* represents an individual study in the meta-analysis, with size of the symbol proportional to the sample size of the study. The *open circle* represent PVS, the *filled upright triangle* represents ASVS, and the *filled square* represents ^18^F-DOPA PET. The Q* statistic represents the point where sensitivity and specificity are equal. *AUC* area under the summary ROC curve, *SE* standard error.

Trials reporting on PVS included 65 patients. Pooled data estimated a sensitivity of 0.87 (95 % CI; 0.70, 0.96), a positive LR of 3.6 (95 % CI; 1.99, 11.9), and a negative LR of 0.23 (95 % CI; 0.11, 0.52). Heterogeneity was present for specificity (*Ι* ^2^ = 30 %, *χ* ^2^ = 4.29, *df* = 3, *p* = 0.23). Graphically, the trial from de Lonlay, et al. [7] stood out (specificity 0.73 vs*.* 1.00). The summary DOR of 23.5 (95 % CI; 6.1, 90.9) was consistent across studies (*Ι* ^2^, 0 %). The summary ROC showed an AUC of 0.90 and a Q* of 0.83. These results were consistent across studies (*Ι* ^2^, 0 %; Fig. 2).

Two trials were identified that reported on ASVS and included 60 patients. Heterogeneity was present for specificity (*Ι* ^2^ = 54 %, *χ* ^2^ = 2.18, *df* = 1, *p* = 0.14) and positive LR (*Ι* ^2^ = 31 %, *χ* ^2^ = 1.46, *df* = 1, *p* = 0.23) estimates. Pooled data estimated a sensitivity of 0.71 (95 % CI; 0.55, 0.84) and a negative LR of 0.93 (95 % CI; 0.26, 0.82). The estimate of the summary DOR was 4.3 (95 % CI; 1.3, 14.7) and was consistent across studies (*Ι* ^2^, 0 %). The summary ROC showed an AUC of 0.73 and a Q* of 0.67. These results were homogenous across studies (*Ι* ^2^, 0 %; Fig. 2).

Trials reporting on ^18^F-DOPA PET included 132 patients. Heterogeneity was observed for sensitivity (*Ι* ^2^ = 50 %, *χ* ^2^ = 12.6, *df* = 6, *p* = 0.06). Graphically, the studies from Hardy et al. [33] and Masue et al. [34] stood out. Both studies underestimated the sensitivity compared to the pooled estimate of the other studies (0.75 and 0.67 vs*.* 0.96). Pooled data estimated a specificity of 1.00 (95 % CI; 0.93, 1.00), a positive LR of 9.49 (95 % CI; 3.5, 26), and a negative LR of 0.23 (95 % CI; 0.14, 0.37). The estimate of the summary DOR was 73.2 (95 % CI; 19.2–297.7) and was consistent across studies (*Ι* ^2^, 0 %). The summary ROC showed an AUC of 0.95 and a Q* of 0.90. These were also consistent across studies (*Ι* ^2^, 0 %; Fig. 2).

### Localization Accuracy

Table 5 shows the localization accuracy of PVS, ASVS and ^18^F-DOPA PET.

**Table 5:** Localization accuracy; in order of relevance and validity

Author (year)	Determinant	*n*	Localization accuracy (95% CI)
Capito et al. (2009) [27]	PVS	35	0.77 (0.60, 0.90)
Crétolle et al. (2002) [28]	PVS	45	0.76 (0.60, 0.87)
Pooled estimate	PVS	80	0.76 (0.65, 0.85)
Stanley et al. (2004) [16]	ASVS	39	0.62 (0.45, 0.77)
Chigot et al. (2001) [31]	ASVS	6	0.83 (0.36, 1.00)
Pooled estimate	ASVS	45	0.64 (0.49, 0.78)
Hardy et al. (2007) [33]	^18^F DOPA PET/CT	24	0.75 (0.53, 0.90)
Zani et al. (2011) [36]	^18^F DOPA PET/CT	15	0.73 (0.45, 0.92)
Capito et al. (2009) [27]	^18^F DOPA PET	16	0.81 (0.54, 0.96)
Barthlen et al. (2008) [32]	^18^F-DOPA PET/CT	10	0.81 (0.55, 1.00)
Ribeiro et al. (2007) [30]	^18^F-DOPA PET/MRI	15	0.90 (0.66, 1.00)
Otonkoski et al. (2006) [35]	^18^F-DOPA PET	5	1.00 (0.48, 1.00)
Pooled estimate	^18^F DOPA PET	84	0.82 (0.72, 0.90)

*n* number of study subject with focal disease that underwent PVS and subsequent surgical intervention; *CI* confidence interval; *CT* computed tomography; *MRI* magnetic resonance imaging; *PVS* pancreatic venous sampling; *ASVS* selective pancreatic arterial calcium stimulation with hepatic venous sampling; ^*18*^ *F-DOPA PET* fluorine-18 l-3,4-dihydroxyphenylalanine positron emission tomography

Trials reporting on PVS included 80 patients. All included studies were at risk for bias, but pooled data showed homogeneity among their results (*Ι* ^2^ = 0 %). Pooled data estimated a localization accuracy of 0.76 (95 % CI; 0.65, 0.85) in detecting the correct location of the focal lesion in the pancreas.

Two studies reported the localisation accuracy of ASVS. Included trials reported on a total of 45 patients. Heterogeneity was observed between included studies (*Ι* ^2^ = 17 %, *χ* ^2^ = 1.20, *df* = 1, *p* = 0.27), but was not regarded as clinically relevant. Pooled data estimated a localization accuracy of 0.64 (95 % CI; 0.49, 0.78).

Trials reporting on ^18^F-DOPA PET included 84 patients. Heterogeneity between study results was small (*Ι* ^2^ = 6 %). Pooled data estimated a localization accuracy of 0.82 (95 % CI; 0.72, 0.90).

## Discussion

CHI is the main cause of persistent hypoglycaemia in infancy and childhood. Early detection and appropriate management is crucial for avoiding neurologic complications. In patients requiring surgery distinguishing focal from diffuse disease can fundamentally change the surgical management to focal curative pancreatectomy and near-total palliative pancreatectomy, respectively. Furthermore, preoperative localisation of focal disease is vital for identifying the focal lesion during curative surgery. Three modalities have been introduced for these purposes: PVS, ASVS and ^18^F-DOPA PET. To establish the diagnostic value of each modality a systematic review and meta-analysis was conducted.

Over 1,400 original publications were identified through a systematic review of the literature. Of these articles, only 13 articles reported original data on the diagnostic and localisation accuracy of PVS, ASVS, and/or ^18^F-DOPA PET in patients requiring surgery for CHI.

Aside from the sensitivity, ^18^F-DOPA PET was superior in every parameter of diagnostic accuracy, including the parameters that are considered most informative in evaluating diagnostic accuracy (i.e. positive LR, negative LR, DOR, AUC, and the Q* statistic; Table 6). Furthermore, ^18^F-DOPA PET was found to be superior in localising focal CHI (pooled localisation accuracy, 0.82) when compared to PVS (pooled localisation accuracy, 0.76) and ASVS (pooled localisation accuracy, 0.64).

**Table 6:** Summary outcome measures

Determinant	Pooled sensitivity (95 % CI)	Pooled specificity (95 % CI)	Pooled positive LR (95 % CI)	Pooled negative LR (95 % CI)	Summary DOR (95 % CI)	AUC (SE)	Q* (SE)	Pooled localization accuracy (95 % CI)
PVS	0.87 (0.70, 0.96)^b^	0.73 (0.52, 0.88)^a^	3.6 (1.99, 6.6)	0.23 (0.11, 0.50)	23.5 (6.09, 90.9)	0.90 (± 0.05)	0.83 (± 0.05)	0.76 (0.65, 0.85)
ASVS	0.71 (0.55, 0.84)	0.69 (0.39, 0.91)^a^	2.2 (0.96, 5.2)^a^	0.46 (0.26, 0.82)	4.3 (1.3, 14.7)	0.73 (± 0.09)	0.67 (± 0.07)	0.64 (0.49, 0.78)
^18^F-DOPA PET	0.75 (0.53, 0.90)^a^	1.00 (0.93, 1.00)^b^	9.49 (3.5, 26)^b^	0.23 (0.14, 0.37)^b^	73.2 (19.2, 279.7)^b^	0.95 (±0.02)^b^	0.90 (±0.03)^b^	0.82 (0.72, 0.90)^b^

*CI* confidence interval; *LR* likelihood ratio; *DOR* diagnostic odds ratio; *AUC* area under the summary ROC curve; *SE* standard error; *PVS* pancreatic venous sampling; *ASVS* selective pancreatic arterial calcium stimulation with hepatic venous sampling; ^*18*^ *F-DOPA PET* fluorine-18 l-3,4-dihydroxyphenylalanine positron emission tomography

^a^Heterogeneity among study results. Summary estimate based on a single study of the highest scientific validity

^b^Values represents the summary outcome estimate with the highest diagnostic accuracy

With an estimated specificity of 1.00 a positive PET scan rules in every patient with focal CHI. The possibility of false-positive results due to F-DOPA accumulation in the gall bladder and alimentary tract were not observed [37]. The astounding specificity of ^18^F-DOPA PET scanning is further supported by reports detecting focal CHI in ectopic pancreatic tissue [38, 39].

However, with an estimated sensitivity of 0.75 (range 0.67–1.00), a negative ^18^F-DOPA PET scan cannot rule out focal CHI in patients requiring surgery. False-negative results can occur due to several reasons, including the inability of small and thin lesions to accumulate sufficient F-DOPA to be visualized. The smallest lesion detected by ^18^F-DOPA PET reported in the literature has measured 5 × 4 mm in diameter [35]. Moreover, focal lesions may be missed near the left kidney, gall bladder, and duodenum due to elimination of F-DOPA through the kidneys, liver, biliary tract, gall bladder, and duodenum, especially when focal lesions are small in size. Localisation accuracy of ^18^F-DOPA PET suffered from similar problems.

Besides being diagnostically more accurate, ^18^F-DOPA PET has some important additional advantages over both PVS and ASVS. Most notably, PET scanning is non-invasive, simple, and remains free of reported complication in the medical literature.

It must be said, however, that ^18^F-DOPA PET is not free of risks. It exposes patients to a small but significant fraction of ionizing radiation, albeit far less than the alternative angiographic methods. The effective dose of 1 minute of abdominal fluoroscopy in paediatrics is estimated to be 1 mSv per minute [40]. Since ASVS and PVS procedures can last up to several hours the accumulative exposure to ionizing radiation is substantial. Radiation dosimetry from combined PET/CT, using 80 mAs and 140 kVp for the CT-scan, is approximately 3 to 5 mSv. The estimated risk of eventual death from radiation-induced malignancy in paediatrics by an effective dose of 10 mSv was one in 1,000 [41, 42]. Intelligent dose reduction based on the principles of as low as reasonably achievable is essential for the safest possible care of children [43].

Developments in the field of nuclear medicine, especially hybrid PET/MRI scanning, could further reduce the radiation burden associated with ^18^F-DOPA PET scanning and should—in the near future—be strived for as the ultimate modality to diagnose and localize CHI [44].

### Limitations

Our systematic review and meta-analysis has several limitations. The meta-analysis reported here combined results from studies with both low and high probability of bias. Furthermore, sample sizes varied greatly among included studies, where the smallest sample included only four patients [29]. Nevertheless, even when incorporating studies with small sample sizes, combining the results of multiple studies increases the diagnostic accuracy of outcome estimates to the levels that are largely unachievable by stand-alone studies [45]. Furthermore, combining results from multiple studies can detect homogeneity among their results making estimated diagnostic accuracy generalizable to other clinics.

Although the majority of included studies reported a clinically applicable domain, the overall methodological quality of papers was generally poor. The most consistent flaw in study methodology was insufficient blinding of the assessor of the reference standard to the outcome of the index test and vice versa. Only three studies reported sufficient blinding protocols [16, 32, 33]. Furthermore, a considerable amount of studies had missing data, either resulting from non-standardized selection of the domain or from a failure to perform the reference standard on all included patients. Studies with small sample sizes and high probability of bias tended to overestimate the diagnostic accuracy and could have attributed to the observed heterogeneity among study results.

Risk of publication bias assessment was considered inappropriate and not meaningful. Application among meta-analysis with small number of studies (*n* < 10) yields low statistical power [46]. Furthermore, large in-between study heterogeneity could lead to false-positive claims of publication bias [47]. Both were applicable to our meta-analysis. Therefore, publication bias assessment was not performed

Despite these limitations, homogeneous study results were observed for most parameters relating to the diagnostic accuracy of ASVS, PVS, and ^18^F-DOPA PET. Therefore, we feel confident that the estimated parameters of diagnostic accuracy approach the levels achieved in a clinical setting. Nonetheless, the low number of studies included, the low number of study subjects, and the poor overall methodological quality of the included studies limit the power of this meta-analysis in providing strong conclusions and recommendations. A well-designed cross-sectional trial investigating a large population of children with CHI should be performed to make stronger conclusions and recommendations about the diagnostic superiority of ^18^F-DOPA PET as claimed by the results of this systematic review and meta-analysis.

## Conclusions

In conclusion, this systematic review and meta-analysis found evidence for the superiority of ^18^F-DOPA PET in diagnosing and localizing focal CHI in patients requiring surgery for this disease. A positive PET scan rules in every patient with focal CHI. A negative PET scan should, however, be approached with caution, because false-negative results tend to occur. As a non-invasive and accurate modality, ^18^F-DOPA PET is poised to replace PVS and ASVS in the diagnostic management of CHI.
